# Spider-derived peptide LCTx-F2 suppresses ASIC channels by occupying the acidic pocket

**DOI:** 10.1016/j.jbc.2025.108286

**Published:** 2025-02-10

**Authors:** Canwei Du, Fuchu Yuan, Zhongzhe Zhang, Ziyan He, Guohao Liu, Wenqian Hou, Meichun Deng, Changjun Liu, Mingqiang Rong

**Affiliations:** 1 School of Life and Health Sciences, Hunan University of Science and Technology, Xiangtan, Hunan, China; 2 The National & Local Joint Engineering Laboratory of Animal Peptide Drug Development, College of Life Sciences, Hunan Normal University, Changsha, Hunan, China; 3 Department of Biochemistry and Molecular Biology & Hunan Key Laboratory of Animal Models for Human Diseases, School of Life Sciences, Central South University, Changsha, China; 4 Frontiers Medical Center, Tianfu Jincheng Laboratory, Chengdu, Sichuan, China

**Keywords:** ASICs, peptide mutation, acidic pocket, inhibition, dynamic simulation

## Abstract

Acid-sensing ion channels (ASICs) are proton-evoked sodium ion channels, highly distributed in the peripheral and central nervous system. ASICs are involved in pain perception, and ASIC3 channel is presumed as the target of promising analgesics. Peptide drugs have attracted the attention of pharmaceutical developers because of their advantages such as low toxic side effects and targeted specificity. Although numbers of chemicals acting on ASICs are emerging, there are limited reports on peptide inhibitor acting on ASIC3 channel. Here, we found that spider-derived peptide LCTx-F2 suppressed the activity of ASIC3 channel in a concentration-dependent manner. By performing peptide mutation and molecular docking, we revealed the molecular mechanism of LCTx-F2 inhibiting ASIC3 channel, in which **β**-hairpin of LCTx-F2 penetrated the acidic pocket of the channel. Similarly, LCTx-F2 also inhibited ASIC1a channel by occupying the acidic pocket, but N terminus of the peptide sticked into the region. The bond relationship between critical residues of LCTx-F2 and the channels was uncovered by molecular docking and dynamic simulation. Thus, our findings indicated the molecular mechanism by which LCTx-F2 acts on ASIC3 and ASIC1a channels and provided a novel template of analgesic drug targeting the channels.

The acid-sensing ion channels (ASICs) are cation channels that are sensitive to amiloride, and they belong to the superfamily of epithelial sodium channels/degenerins (1, 2). ASICs are found in various parts of the body, including the peripheral and central nervous systems, skin, and internal organs (3, 4). These channels play important roles in a wide range of physiological processes, such as ischemia (5), drug addiction (6), mechanosensation (7), alcohol intoxication (8), neurotransmission (9), cancer metastasis (10, 11), and pain perception (12). In mammals, ASICs are composed of seven different subunits encoded by five ASIC genes. These subunits can come together to form either homotrimers or heterotrimers. The structure of ASICs, as revealed by crystal studies, consists of extracellular and transmembrane domains. Each subunit contains distinct domains known as finger, thumb, knuckle, and palm (13, 14, 15). The activation and desensitization of ASICs upon extracellular acidification are associated with conformational changes in the "acidic pocket" (16). Further structural investigations have shown that protonation of the acidic residue in the "acidic pocket" leads to a transition from an expanded conformation in the closed state to a contracted conformation in the open and desensitized states (17, 18, 19, 20). The pore region, formed by the transmembrane α-helices of the three subunits, is involved in channel gating and selectivity (21). The "wrist" domain, which connects the extracellular domain and the pore region, plays a critical role in channel gating (13). As their name suggests, ASICs exhibit proton dependence for channel opening, with half-maximal activation values of approximately 6.5 for ASIC1a and ASIC3, 6.1 for ASIC1b, and 4.5 for ASIC2a (12, 21, 22). Upon activation by extracellular protons, ASICs enter a desensitized state. The structure of chicken ASIC1a has revealed that the β11–β12 linkers in the palm domain act as a molecular "clutch", facilitating rapid channel desensitization (17).

There is increasing evidence suggesting that ASICs can be influenced by both endogenous and exogenous chemicals. ASIC channels, particularly ASIC1a and ASIC3, are affected by certain cations like Ca^2+^ and Zn^2+^, which provides valuable insights for the development of novel drugs targeting ASICs. Insulin has been found to regulate the trafficking of various ion channels, including ASIC1a. Depletion of insulin leads to an increase in ASIC1a channel expression in Chinese hamster ovary cells and neurons. In addition, dynorphin opioid peptides have been shown to prevent the desensitization of ASIC1a channels, thereby enhancing ischemic brain injury. Protease treatment has been found to impact the activity of ASIC1a channels, which play a role in brain injury. For example, the cleavage of the channel by matriptase reduces its current in *Xenopus oocytes*. Proinflammatory mediators like nerve growth factor and bradykinin can induce the excitation of nociceptive fibers and increase the expression of ASICs in dorsal root ganglion neurons. In addition to proton sensing, ASIC3 channels can be activated by a nonproton ligand called 2-guanidine-4-methylquinazoline, which interacts with the extracellular palm domain (23). Amiloride, a nonselective inhibitor acting on the epithelial sodium channel superfamily, can suppress most ASICs, and it has shown analgesic effects in humans and provides protection against acidosis-induced neuron damage. Certain peptide toxins derived from animal venoms, such as APETx2 from sea anemone, mambalgin from mamba snakes, MitTx from coral snakes, and PcTx1 from spiders, have been found to target ASICs (24). Mambalgin toxins inhibit ASICs by binding to the thumb domain, exhibiting analgesic effects in animal models (14, 25, 26). MitTx, on the other hand, activated the proton-exciting currents of ASICs, leading to pain. The X-ray structure of MitTx has revealed its interaction with the wrist and thumb domains of the channels (18, 27). PcTx1, an inhibitory cysteine knot toxin, inserts into the acidic pocket of ASIC1a channels and exhibits neuroprotection in ischemic stroke (15, 28).

Venom serves as a crucial weapon for animals in hunting prey and defending against enemies. Spider venom contains a variety of peptide toxins, some of which induce pain by targeting different ion channels (29, 30, 31), whereas others have analgesic properties and block the pain pathway (32, 33, 34). For instance, Hi1a from the funnel-web spider *Hadronyche infensa* and PcTx1 from the Trinidad Chevron tarantula *Psalmopoeus cambridgei* selectively inhibit the ASIC1a channel but not the rASIC2a or rASIC3 channels (35, 36). Derived from the spider *Lycosa singoriensis*, LCTx-F2 enhances blood coagulation by targeting coagulation factors like FXIIa and thrombin, without causing cytotoxicity or hemolysis (37, 38). Here, we discovered that LCTx-F2 inhibits the rASIC3 and rASIC1a channels in a concentration-dependent manner. Through peptide mutation and molecular docking, we found that LCTx-F2 inserts into the acidic pocket of the rASIC3 and rASIC1a channels in different orientations. By combining these findings with dynamic simulation, we gained insights into the bond relationship between LCTx-F2 and the two channels. Our study revealed that the coagulation-potentiating peptide LCTx-F2 inhibited the activity of rASIC3 and rASIC1a channels by occupying the acidic pocket.

## Results

### LCTx-F2 inhibited rASIC3 channel

**Figure 1:**
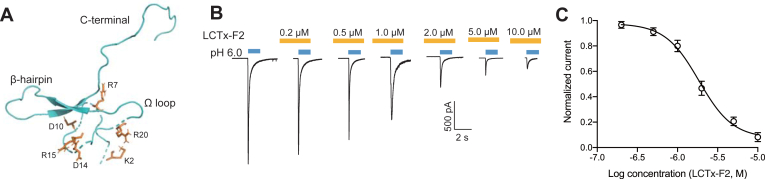
**LCTx-F2 inhibited rASIC3 channel**. *A*, the predicated structure of LCTx-F2. β-hairpin, Ω loop, and C terminus of LCTx-F2 were labeled. Some residues in LCTx-F2 are shown in *stick* representation. *B* and *C*, representative current traces (*B*) and the effect and concentration (C) of LCTx-F2 inhibiting rASIC3 channel evoked by proton. After 80 s perfusion of LCTx-F2 in pH 7.4 solution, the current of rASIC3 channel was elicited by proton in (*B*). The Hill equation was employed to fit the data in (*C*) (n = 4–5). ASIC, acid-sensing ion channel.

LCTx-F2, derived from spider venom, has been found to promote coagulation by targeting coagulation factors, and some crucial residues in LCTx-F2 were discovered to affect its function on potentiating coagulation factors. The spatial structure of LCTx-F2 was obtained *via* SWISSMODEL (https://swissmodel.expasy.org/) with purotoxin-2 (Protein Data Bank [PDB] ID: 2MZF) as a template (Fig. 1*A*) (38). Recently, we observed that LCTx-F2 showed a strong affinity for the rASIC3 channel. When we perfused to the rASIC3 channel with LCTx-F2, the sodium current evoked by protons was significantly reduced, and at a concentration of 10.0 μM, the current was almost completely abolished (Fig. 1*B*). Furthermore, we found that LCTx-F2 inhibited the rASIC3 channel in a concentration-dependent manner, with an IC_50_ of 1.85 μM ± 0.51 μM and a Hill Slope of 2.06 ± 1.18 (Fig. 1*C*). Thus, our study revealed that the spider-derived peptide LCTx-F2 inhibited rASIC3 channel in a dose-dependent manner.

### Mutations in LCTx-F2 affected its affinity to rASIC3 channel

**Figure 2:**
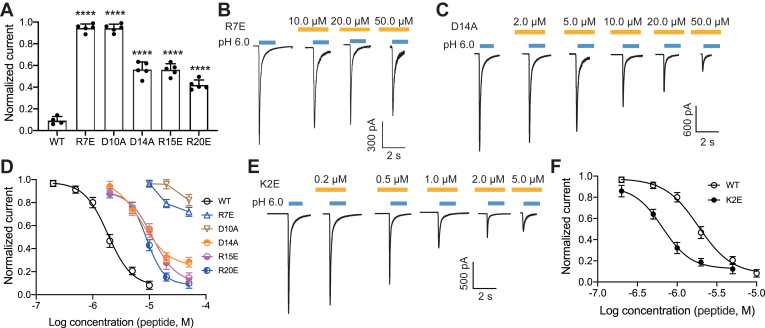
**The effect of mutants of LCTx-F2 on rASIC3 channel**. *A*, the averaged inhibitory effect of 10.0 μM mutants of LCTx-F2 on rASIC3 channel. The current of rASIC3 channel was elicited by proton (pH 6.0 solution). The data were represented as the mean ± SD. ∗∗∗∗*p* < 0.0001, performed by one-way ANOVA followed by Dunnett’s *post hoc* test, significantly different of mutant group compared with WT group (n = 3). *B*, *C*, and *E*, the representative traces of mutants R7E (*B*), D14A (*C*), and K2E (*E*) inhibiting rASIC3 channel evoked by proton. *D*, the dose–response of some mutants of LCTx-F2 inhibiting rASIC3 channel as compared with WT of the receptor (n = 4). *F*, the dose–response of mutant K2E inhibiting rASIC3 channel as compared with WT of the receptor (n = 4). ASIC, acid-sensing ion channel.

In our previous findings, we observed that mutations in specific residues of LCTx-F2 resulted in a decrease in its coagulation-promoting activity on thrombin and FXIIa (38). To further investigate the mechanism of LCTx-F2's inhibition on rASIC3 channel, we conducted patch-clamp recordings to assess the effects of certain LCTx-F2 mutants on the channel. Both mutants R7E and D10A, at a concentration of 10.0 μM, showed a tiny inhibitory activity on the rASIC3 channel (Fig. 2*A*), displaying significant differences compared with that of WT LCTx-F2 on the channel. Even at a higher concentration (50.0 μM), only 28.1% and 18.9% of rASIC3 channel activity were reduced by mutants R7E and D10A, respectively (Fig. 2, *B* and *D*). On the other hand, mutations in residues Asp14, Arg15, and Arg20 displayed a moderate effect on the affinity of LCTx-F2 to the rASIC3 channel (Fig. 2*A*). Mutants D14A, R15E, and R20E of LCTx-F2 still exhibited inhibitory activity on the channel, with IC_50_ values of 7.46 μM ± 1.53 μM, 11.33 μM ± 4.07 μM, and 8.94 μM ± 1.91 μM, respectively (Fig. 2, *C* and *D*). Interestingly, glutamate substitution in residue Lys2 of LCTx-F2 enhanced its activity on the rASIC3 channel, and the mutant K2E showed a higher affinity to the channel with an IC_50_ of 0.66 μM ± 0.43 μM, compared with the WT LCTx-F2 (Fig. 2, *E* and *F*). Therefore, some residues' replacement in peptide LCTx-F2 would affect its activity on the rASIC3 channel.

### LCTx-F2 binding to rASIC3 channel

**Figure 3:**
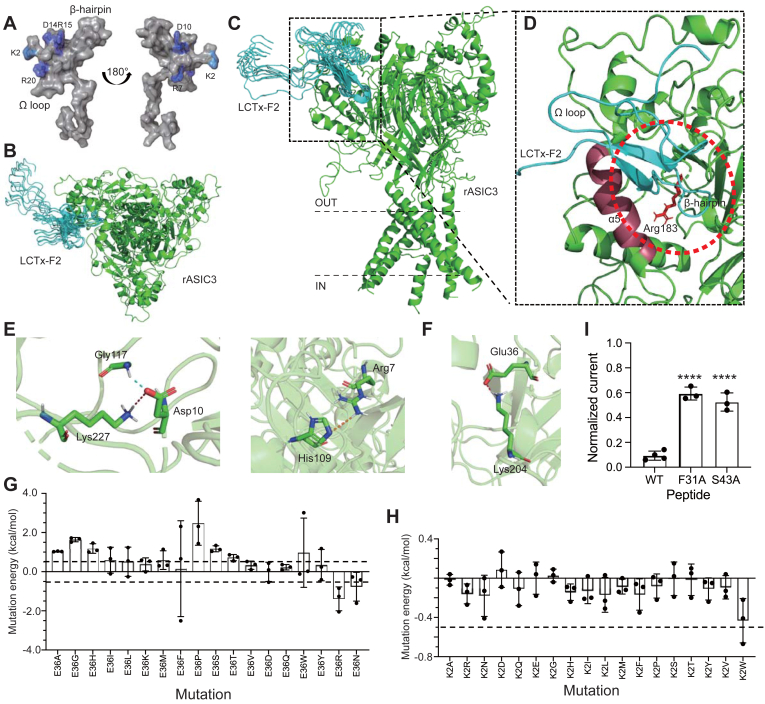
**LCTx-F2 binding to rASIC3 channel**. *A*, surface model of LCTx-F2 with the basic skeleton. β-hairpin and Ω loop of LCTx-F2 were labeled. Residues Lys2, Arg7, Asp10, Asp14, Arg15, and Arg20 were colored by *blue*. *B* and *C*, *top view* (*B*) and *side view* (*C*) of LCTx-F2 binding to rASIC3 channel. LCTx-F2 and rASIC3 channel were colored by *cyan* and *green*, respectively. *D*, close-up view of LCTx-F2 inserted into the acidic pocket of rASIC3 channel. LCTx-F2 were colored by *cyan*. Residue Arg183 in rASIC3 channel are shown in *stick* representation. Acidic pocket is shown by *red circle*, and helix α5 is colored *raspberry*. *E*, bond relationship of Asp10 (*left*) and Arg7 (*right*) in LCTx-F2 with rASIC3 channel. Asp10, Arg7 of LCTx-F2 and Gly117, Lys227, His109 of ASIC3 channel are shown in *stick* representation. Salt bridge, pi interaction, and hydrogen bond were colored by *red*, *orange*, and *cyan*, respectively. *F*, bond relationship of Glu36 in LCTx-F2 with rASIC3 channel. Salt bridge is colored *red*. *G* and *H*, saturation mutation energy of residues Glu36 (*G*) and Lys2 (*H*) in LCTx-F2 binding to rASIC3 channel. Mutation energy > +0.5 kcal/mol was considered as significantly different. *I*, the averaged inhibitory effect of 10.0 μM mutants of LCTx-F2 on rASIC3 channel. The current of rASIC3 channel was elicited by proton (pH 6.0 solution). The data were represented as the mean ± SD. ∗∗∗∗*p* < 0.0001, performed by one-way ANOVA followed by Dunnett’s *post hoc* test, significantly different of mutants group compared with WT group (n = 3). ASIC, acid-sensing ion channel.

The patch-clamp recording results revealed that mutations in residues Arg7 and Asp10 of LCTx-F2 significantly reduced its inhibitory activity on the rASIC3 channel. To gain further insights into the mechanism of LCTx-F2 in inhibiting the rASIC3 channel, we conducted molecular docking to examine the binding of LCTx-F2 to the channel, with residues Arg7 and Asp10 of LCTx-F2 serving as the ligand-binding sites. Since the crystal structure of the rASIC3 channel is unavailable, we generated a structural model of the channel using SWISS-MODEL (https://swissmodel.expasy.org/), with the human ASIC1a channel (PDB ID: 7CFS) (25) as a template. After refining the docking, we obtained 100 poses from a total of 2000 poses. Considering the patch-clamp recording results, we identified a 10-pose cluster that represents the most probable binding model of LCTx-F2 with the rASIC3 channel. This model suggested that LCTx-F2 may bind to the extracellular side of the channel and inserted into the acidic pocket (Fig. 3, *B* and *C*). Specifically, the β-hairpin of LCTx-F2 penetrated the acidic pocket, whereas the N terminus, including residue Lys2, exhibited a weak connection with the receptor (Fig. 3*D*). Similar to the role of its C terminus in coagulation factors, the C terminus of LCTx-F2 showed minimal interaction with the rASIC3 channel (Fig. 3*C*).

To understand how LCTx-F2 inhibits the rASIC3 channel, we analyzed the bond relationship between key residues in LCTx-F2 and the receptor. We found that a mutation in residue Asp10 completely eliminated its inhibitory activity on the rASIC3 channel. This residue may form a salt bridge with Lys227 or connect with Gly117 through a hydrogen bond with the backbone nitrogen atom (Fig. 3*E* and table 1). The interaction between Arg7 of the peptide and His109 of the rASIC3 channel, through pi–cation interaction or hydrogen bond, is crucial for its function (Fig. 3*E* and table 1). Several residues in the β-hairpin of LCTx-F2 were found to have a strong connection with the rASIC3 channel. For example, residue Glu36 of LCTx-F2 may form a salt bridge with residue Lys204 located in the acidic pocket of the rASIC3 channel (Fig. 3*F*). Although we could not obtain mutants of residue Glu36 in LCTx-F2 (38), we performed a virtual saturation mutation using Discovery Studio 2019 (BIOVIA) to evaluate the effect of this residue on its affinity to the rASIC3 channel. Half mutation in residue Glu36 of LCTx-F2 would weaken its connection with the channel. Importantly, mutation of Glu36 to Asp had little impact on the binding energy (Fig. 3*G*). In comparison, mutations in residue Lys2 had minimal effect on the connection between the peptide and the rASIC3 channel (Fig. 3*H*). This is consistent with the result of patch-clamp recording, which showed that the affinity mutant K2E to the rASIC3 channel only increased threefold compared with LCTx-F2. Other residues, such as Ser43 or Phe31 in LCTx-F2, may also affect its activity on the rASIC3 channel (Table 1). Patch-clamp recording results indicated that mutations in residues Phe31 or Ser43 of LCTx-F2 reduced its inhibitory activity on the rASIC3 channel (Fig. 3*I*). Therefore, we discovered that the β-hairpin of LCTx-F2 inserted into the acidic pocket of the rASIC3 channel, and the interaction between critical residues in the peptide and its receptors supported this connection.

**Table 1:** Bond relationship of residues in LCTx-F2 with rASIC3

Residue	Bond relationship	Bond type	Distance
ASP10	ASP10:OD2 - B: LYS227:HZ2	Salt bridge	3.25
	ASP10:OD2 - B: GLY117:HN	Conventional	2.02
ARG7	ARG7:HH12 - B: HIS109	Pi–cation	2.89
	ARG7:HH22 - B: HIS109:O	Conventional	2.80
GLU36	GLU36:OE2 - C: LYS204:HZ1	Salt bridge	1.90
	GLU36:O - C: ASN209:HD22	Conventional	2.01
ASP37	ASP37:O - C: ASN209:HD22	Conventional	2.66
SER43	A: SER43:O - B: LYS350:HZ2	Conventional	2.20
PHE31	PHE31 - B: LYS350:CG	Pi–sigma	3.92

### LCTx-F2 also inhibited rASIC1a channel

**Figure 4:**
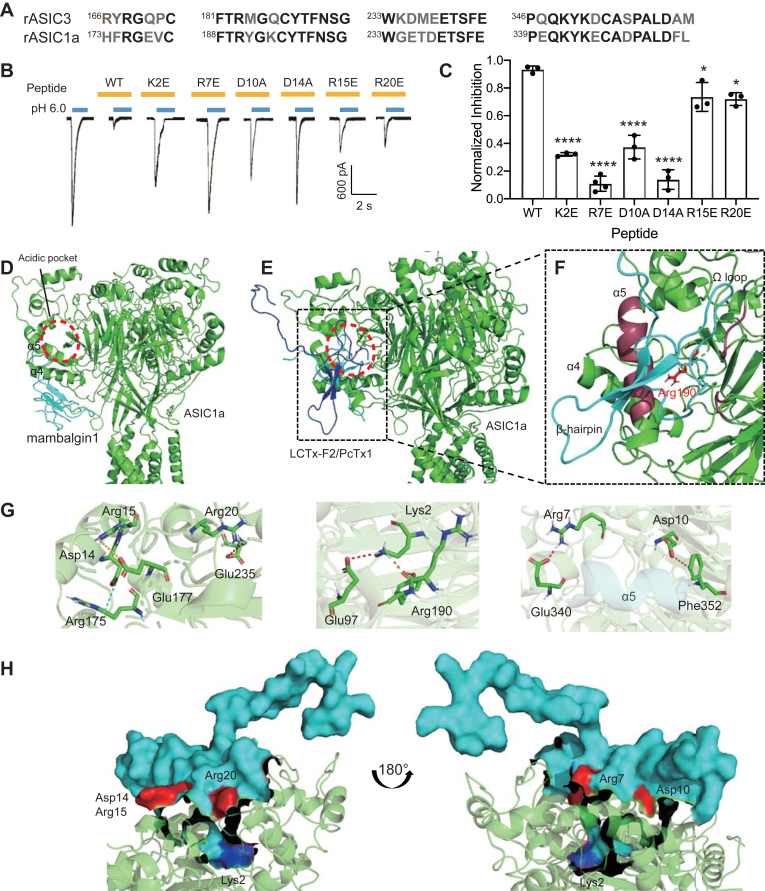
**LCTx-F2 inhibiting rASIC1a channel**. *A*, the sequence alignment of rASIC3 channel and rASIC1a channel at the acidic pocket is shown. The same residues both in rASIC3 channel and rASIC1a channel are indicated in *black*. *B* and *C*, representative current traces (*B*) and the averaged inhibitory effect (*C*) of 10.0 μM WT or mutants of LCTx-F2 inhibiting rASIC3 channel evoked by proton. After 80 s perfusion of LCTx-F2 in pH 7.4 solution, the current of rASIC3 channel was elicited by proton in (*B*). The data (*C*) were represented as the mean ± SD. ∗∗∗∗*p* < 0.0001 and ∗ *p* < 0.05 (R15E, 0.0195 and R20E, 0.0114), performed by one-way ANOVA followed by Dunnett’s *post hoc* test, significantly different of mutant group compared with WT group, respectively (n = 3). *D*, side view of snake toxin mambalgin1 binding to hASIC1a channel (Protein Data Bank ID: 7CFT). Toxin mambalgin1 and ASIC1a channel were colored as *cyan* and *green*, respectively. *E*, side view of toxins LCTx-F2 and PcTx1 binding to hASIC1a channel. LCTx-F2, PcTx1, and ASIC1a channel were colored as *blue*, *cyan*, and *green*, respectively. *F*, close-up view of LCTx-F2 inserted into the acidic pocket of ASIC1a channel. LCTx-F2 were colored by *cyan*. Residue Arg190 in ASIC1a channel is shown as *sticks*. Acidic pocket is shown by *red circle*, and helix α5 is colored *raspberry*. Asp14, Arg15, Arg20, Lys2, Arg7, Asp10 of LCTx-F2 and Arg175, Glu177, Glu235, Glu97, Arg190, Glu340, Phe352 of ASIC1a channel are shown as *stick*. *G*, bond relationship of some critical residues in LCTx-F2 with rASIC3 channel. Salt bridge, pi interaction, and hydrogen bond are colored by *red*, *orange*, and *cyan*, respectively. *H*, model of LCTx-F2 binding to ASIC1a channel. LCTx-F2 is shown in surface. Lys2 in LCTx-F2 is colored in *blue*, and some critical residues is colored by *red*. ASIC, acid-sensing ion channel.

Considering that rASIC1a channel shared a similar sequence to ASIC3 channel, especially in the acidic pocket (Fig. 4*A*), we also investigated the effect of LCTx-F2 on the rASIC1a channel. LCTx-F2 exhibited potent inhibition of the rASIC1a channel, with 10.0 μM LCTx-F2 suppressing over 90% of the current evoked by protons (Fig. 4, *B* and *C*). We further examined the effect of certain mutants of LCTx-F2 on the rASIC1a channel. Mutants R7E and D14A almost completely abolished its inhibitory activity on the channel, whereas mutations in residues Arg15 and Arg20 of LCTx-F2 showed only a slight change in affinity to the rASIC1a channel (Fig. 4, *B* and *C*). In contrast to the higher inhibition observed in mutant K2E on the rASIC3 channel, mutant K2E exhibited lower inhibitory activity on the rASIC1a channel compared with the WT peptide (Fig. 4, *B* and *C*). Mutation in residue Asp10 of LCTx-F2 also resulted in a moderate decrease in its inhibition of the rASIC1a channel (Fig. 4, *B* and *C*). Therefore, LCTx-F2 also effectively suppressed the rASIC1a channel, and certain mutations in critical residues of the peptide reduced its inhibitory effect.

To understand the mechanism of LCTx-F2 inhibiting the rASIC1a channel, we performed a molecular docking analysis. Since the human ASIC1a channel shares a high sequence homology with the rat ASIC1a channel, we used the structure of the human ASIC1a channel (PDB ID: 7CFS) for the docking. The residues Arg7 and Asp14 in LCTx-F2 were considered as the ligand-binding sites, and considering the probable bond relationship between LCTx-F2 and ASIC1a channel, only a pose was obtained. Previous studies have shown that snake toxin mambalgin-1 reduces the proton sensitivity of the hASIC1a channel by interacting with helix α5 of the thumb domain (Fig. 4*D*) (25). However, in the case of LCTx-F2, it appears to bind to the acidic pocket of the ASIC1a channel, similar to the mechanism of toxin PcTx1 inhibiting the channel (Fig. 4*E*) (15, 19). Unlike the β-hairpin of LCTx-F2 that penetrated the acidic pocket of the rASIC3 channel, the N terminus of the peptide reached into the acidic pocket of the ASIC1a channel, and the Ω loop of LCTx-F2 may play an important role in the interaction with the receptor (Fig. 4*F*).

To further investigate the mechanism by which LCTx-F2 inhibits the rASIC1a channel, we conducted an analysis of the bond relationship between key residues in LCTx-F2 and the rASIC1a channel. First, it is possible that Lys2 of LCTx-F2 forms a salt bridge with Glu97 of the ASIC1a channel or interacts with Arg190 through a hydrogen bond (Fig. 4*G* and Table 2). In addition, residues Asp14, Arg15, and Arg20 in LCTx-F2 may establish connections with Arg175, Glu177, and Glu235 of the ASIC1a channel, respectively. Furthermore, Arg7 and Asp10 of LCTx-F2 may interact with Glu340 and Phe352 in helix α5 of the channel (Fig. 4*G* and Table 2). Importantly, residues such as His9 and His13 in LCTx-F2 also exhibited interactions with helix α5 of the ASIC1a channel (Table 2). Overall, LCTx-F2 inhibits the ASIC1a channel by having the residue Lys2 bind to the acidic pocket of the channel, whereas the intermediate section of the peptide acts as a lid, held in place by critical residues (Fig. 4*H*).

**Table 2:** Bond relationship of residues in LCTx-F2 with rASIC1a channel

Residue	Bond relationship	Bond type	Distance
Lys2	LYS2:HZ3 - C: GLU97:OE2	Salt bridge	3.14
	LYS2 - C: TYR191	Pi–alkyl	4.50
	LYS2:HZ2 - C: ARG190:O	Conventional	2.09
ARG7	ARG7:HH12 - C: GLU340:OE2	Salt bridge	1.95
ASP10	ASP10:OD2 - C: PHE352	Pi–anion	3.29
ASP14	ASP14:OD1 - A: ARG175:CD	Carbon	3.50
ARG15	ARG15:HN - A: GLU177:OE2	Conventional	2.52
ARG20	ARG20:HH11 - C: GLU235:OE2	Salt bridge	2.69
	ARG20:CD - C: GLU235:OE2	Carbon	3.49
GLU22	GLU22:OE2 - A: LYS392:HZ2	Salt bridge	2.94
HIS9	HIS9 - C: ASP347:OD2	Pi–anion	3.80
HIS13	HIS13 - C: ASP351:OD2	Pi–anion	3.33
	HIS13:HD1 - C: ASP351:OD1	Conventional	2.24

### Dynamic simulation of LCTx-F2 inhibiting ASIC channels

**Figure 5:**
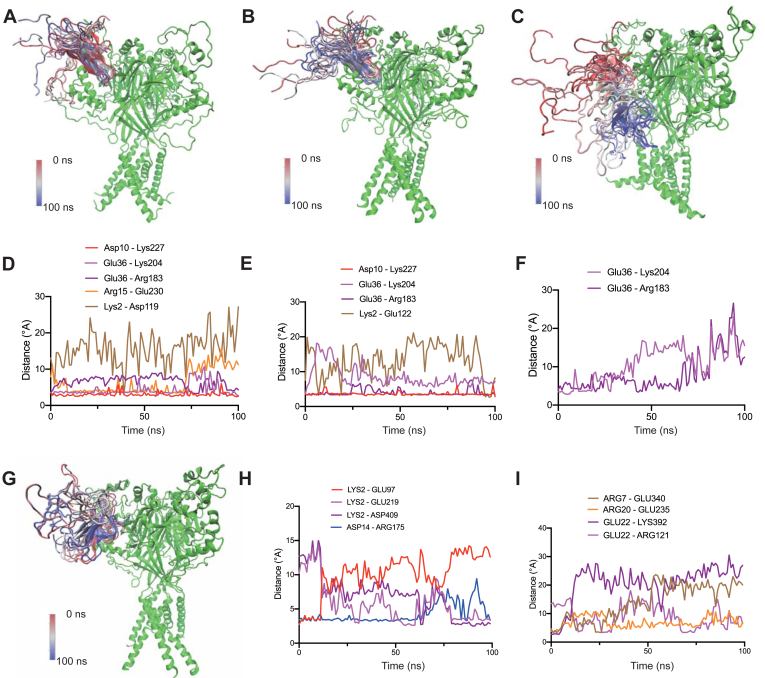
**Dynamic simulation analysis of LCTx-F2 binding to ASIC3 channel or ASIC1a channel**. *A*–*C*, three poses of rASIC3 channel and superimposed LCTx-F2′s conformation during dynamic simulation of 100 ns (10 frames, 10 ns/frame). For clarity, only a representative rASIC3 channel structure from a single frame is shown in *green*. *D*–*F*, salt bridges between residues of LCTx-F2 and rASIC3 channel during simulation of 100 ns, coming from the dynamic simulation (*A*–*C*). *G*, representative pose of hASIC1a channel and superimposed LCTx-F2′s conformation during dynamic simulation of 100 ns (10 frames, 10 ns/frame). For clarity, only a representative hASIC1a channel structure from a single frame is shown in *yellow*. *H* and *I*, salt bridges between residues of LCTx-F2 and hASIC1a channel during simulation of 100 ns, coming from the dynamic simulation (*G*). ASIC, acid-sensing ion channel.

Considering the limitations of rigid molecular docking, dynamic simulations were conducted to provide a more comprehensive analysis of the binding interactions between LCTx-F2 and the ASIC3 or ASIC1a channels. Three representative poses of LCTx-F2 inhibiting the ASIC3 channel were selected for further dynamic simulations. The two poses showed β-hairpin of LCTx-F2 solidly inserted into the acidic pocket of the channel in the simulation of 100 ns (Fig. 5, *A* and *B*), indicating that LCTx-F2 built a stable interaction with ASIC3 channel. We also observed that LCTx-F2 might escape from the acidic pocket of ASIC3 channel in the third pose (Fig. 5*C*). The dynamic simulation showed that C terminus of the peptide displayed little role for its binding to the receptor (Fig. 5, *A* and *B*), which is consistent with the result of molecular docking (Fig. 3*C*). Through molecular docking, we identified that residue Asp10 of LCTx-F2 might form a salt bridge with residue Lys227 of the ASIC3 channel (Fig. 3*E*). More data showed that Asp10 of the peptide kept the close connection with Lys227 of the receptor in the simulation of 100 ns (Fig. 5, *D* and *E*). Residue Glu36 in LCTx-F2 was presumed to go deep into the acidic pocket of ASIC3 channel and might contact Lys204 by ionic bond (Fig. 3*F*), which was evidenced by the simulation analysis (Fig. 5, *D* and *E*). We also found that interaction between residue Glu36 and Arg183 of the receptor may enhance their affinity (Fig. 5, *D* and *E*). Mutation in residue Lys2 of LCTx-F2 slightly affected its inhibition on ASIC3 channel (Fig. 2, *E* and *F*), and the residue also kept farther distance from the receptor in the simulation of 100 ns (Fig. 5, *D* and *E*). The third pose might not reflect the binding of LCTx-F2 to its receptor, and the bond analysis also suggested the peptide escape from the acidic pocket of ASIC3 channel (Fig. 5*F*). We also performed dynamic simulation to analyze the binding of LCTx-F2 to ASIC1a channel. Considering consistent with the molecular docking, the N terminus of LCTx-F2 retained a tight connection with the acidic pocket of ASIC1a channel in the simulation of 100 ns (Fig. 5*G*). Compared with that residue, Lys2 of LCTx-F2 exerted tiny effect on its binding to ASIC3 channel, the residue built long-lasting connection with ASIC1a channel. Especially, in the initial binding, the salt bridge connecting Lys2 of LCTx-F2 with residue Glu97 of ASIC1a channel may be crucial; however, the interaction was substituted by the interaction of Lys2 with residue Glu219 or Asp409 of ASIC1a channel (Fig. 5*H*). The ionic bond linking Asp14 of LCTx-F2 with residue Arg175 of ASIC1a channel was persistent in the simulation of 100 ns (Fig. 5*H*), which was consistent with the result of patch-clamp recording (Fig. 4, *B* and *C*). Some linkages between the peptide and ASIC1a channel were also detected by dynamic simulation analysis, like salt bridge happening on residue Arg7 or Arg20 of LCTx-F2, reflecting that mutants R7E and R20E of LCTx-F2 exerted a lower affinity to ASIC1a channel compared with that of WT (Fig. 4, *B* and *C*). Therefore, we further elucidated the molecular mechanism by which LCTx-F2 inhibits the ASIC3 or ASIC1a channel through dynamic simulations.

## Discussion

ASICs play a crucial role in pain perception among mammals (39, 40). Some animal toxins that are involved in prey capture and defense mechanisms act on ASICs (26, 41). For instance, the venom from *Micrurus tener tener* causes intense and persistent pain by enhancing the activation of ASICs through the MitTx toxin (27). ASICs are also considered important targets for pain relief, and certain chemicals that block their activity have shown strong analgesic effects in tests with living organisms (42, 43). One such chemical is APETx2, which has demonstrated an IC_50_ of 175 nM against the human ASIC3 channel (44). In the study, we discovered that the peptide LCTx-F2, derived from spiders, inhibited the activity of both rat ASIC3 and ASIC1a channels (Figs. 1 and 4*B*). LCTx-F2 belongs to the CSTX family of peptides and possesses a knottin domain (37). Many peptides in this family are believed to interact with ion channels. For example, CsTx-1 can inhibit L-type Ca^2+^ channels (45), whereas purotoxin-2 affects human P2RX3 purinoceptors (46). The inhibition of ASIC3 and ASIC1a channels by LCTx-F2 suggests that neurotoxic peptides from the CSTX family may have a broader role in the survival of spiders. Although several peptides from animal venom have shown affinity for ASICs, only APETx2, derived from sea anemones, has been reported to inhibit both rASIC3 and hASIC3 channels (24). Notably, the structure of LCTx-F2 is distinct from that of APETx2, indicating that the knottin domain in LCTx-F2 could serve as a novel template for designing drugs that target the ASIC3 channel. In addition, LCTx-F2 also inhibits the rASIC1a channel in a similar fashion to the toxin PcTx1, which occupies the acidic pocket of the rASIC1a channel (Fig. 4*E*). However, the two toxins only share 33% homology. Therefore, LCTx-F2 holds promise as a new template for the development of drugs that inhibit ASICs.

To understand how LCTx-F2 inhibits rat ASIC3 and ASIC1a channels at the molecular level, we conducted peptide mutation and molecular docking experiments. When we mutated the Arg7 and Asp10 residues of LCTx-F2, the inhibitory activity on ASIC3 and ASIC1a channels was significantly reduced (Figs. 2*D* and 4, *B* and *C*). Interestingly, the D14A mutant showed minimal function on ASIC1a channel but still exhibited moderate inhibition on ASIC3 channel (Figs. 2*C* and 4, *B* and *C*). Based on the results of electrophysiological recordings, we performed molecular docking and dynamic simulation to investigate the interaction between LCTx-F2 and ASIC3 or ASIC1a channel. We found that LCTx-F2 occupied the acidic pocket of both channels (Figs. 3*C*, 4*E*, and 5, *A*–*C* and *G*), similar to how PcTx1 acts on ASICs (47). This suggests that LCTx-F2 modulates the proton activation of the receptors. In addition, preapplication of LCTx-F2 blocked the proton-evoked currents of ASIC3 or ASIC1a channel. However, when LCTx-F2 was coincubated with a proton solution, it did not inhibit the channels. These results support the idea that LCTx-F2 modulates the proton gating of ASICs, similar to how toxins PcTx1 or mambalgins inhibit ASICs (25, 47). The conformational changes in the acidic pocket of ASICs are known to induce channel activation and desensitization (16). Molecular docking analysis revealed that the β-hairpin of LCTx-F2 penetrates the acidic pocket of ASIC3 channel (Fig. 3*D*), whereas the N terminus of LCTx-F2 sticks into the acidic pocket of ASIC1a channel (Fig. 4*F*). These findings were further supported by patch-clamp recording, which showed that certain residues of LCTx-F2, including Asp10 and Arg7, influence its inhibitory activity on the two channels. Bond relationship analysis showed that these residues build connection with the region near by the acidic pocket of ASIC3 or ASIC1a channel *via* salt bridge or hydrogen bond (Figs. 3*E*, 4, *G* and *H*, 5, *D*–*F*, *H*, and *I*, Tables 1, and 2). We also discovered that the K2E mutant displayed increased affinity for ASIC3 channel (Fig. 2, *E* and *F*) but decreased its inhibitory activity on ASIC1a channel (Fig. 4, *B* and *C*). Residue Lys2 in LCTx-F2 may form a salt bridge with residue Glu97, located in the acidic pocket of ASIC1a channel (Figs. 4*G* and 5*H*). On the other hand, residue Glu36 in the β-hairpin of the peptide may strongly interact with residue Lys204 or Arg193 of ASIC3 channel (Fig. 3*F* and 5, *D*–*E*). Unfortunately, we were unable to obtain mutants to test their affinity to the channel, but mutation energy analysis indicated that changes in residue Glu36 affected the interaction of LCTx-F2 with ASIC3 channel (Fig. 3*G*). When residues Arg7 and Asp14 in LCTx-F2 were set as the crucial sites, only one pose was obtained to perform the dynamic simulation of LCTx-F2 inhibiting ASIC1a channel (Figs. 4*E* and 5*G*). However, it is important to note that additional replicate MD simulations for longer durations may uncover alternative state/interaction possibilities. Our findings suggest that LCTx-F2 blocks the acidic pocket of ASIC3 or ASIC1a channel in different orientations, possibly because of the relatively low homology (55%) between ASIC3 and ASIC1a channels. It is worth noting that some toxins from animal venom have shown similar affinities to different subtypes of the same family of receptors. For example, SsTx can block both KCNQ and Kv1.3 channels (48, 49). Although we used the structural model of the rASIC3 channel with the human ASIC1a channel as a template because of the unavailability of the crystal structure of the rASIC3 channel, our series of experiments have provided valuable insights into the molecular mechanism underlying LCTx-F2's inhibition of ASIC3 and ASIC1a channels.

In our previous results, we observed that LCTx-F2 shortened the coagulation time by affecting coagulation factors (37). In addition, its affinity to ASIC channels suggested that LCTx-F2 may possess a novel bifunctional peptide. It is noteworthy that other peptides derived from animal venom have also demonstrated bifunctional activity. For instance, ShPI-1 from the sea anemone *Stichodactyla helianthus* is known to inhibit serine proteases and voltage-gated potassium channels (50). By conducting peptide mutation, molecular docking, and dynamic simulation, we found that LCTx-F2 potentiates the coagulation factors thrombin and FXIIa in a similar manner (38). The N terminus of the LCTx-F2 peptide binds to the active site cleft of the receptors, whereas specific residues strengthen the connection. We also discovered a similar mechanism for LCTx-F2 in inhibiting ASIC3 and ASIC1a channels. Our study revealed that LCTx-F2 occupies the acidic pocket of the channels, thereby affecting their proton activation. Specifically, the β-hairpin of LCTx-F2 penetrates the acidic pocket of the ASIC3 channel, whereas the N-terminal region holds the region when LCTx-F2 inhibits the ASIC1a channel. The C terminus of LCTx-F2 does not appear to significantly impact its affinity to the coagulation factors, as validated by the truncated mutant T-F2 (38). Although we did not investigate the effect of the mutant T-F2 on ASICs, molecular docking and dynamic simulation indicate that the C terminus of LCTx-F2 has limited interaction with the two channels (Figs. 3*B*, 4*E*, and 5, *A*–*C* and *G*). Consequently, we deduced that the C terminus of LCTx-F2 plays a minor role in its interaction with the receptors. Overall, our findings suggest that LCTx-F2 interacts with its receptors in a similar manner, however, differentiating residues within the peptide govern the interaction.

ASICs play a crucial role in pain perception and are considered promising targets for pain relief. In our study, we investigated the effects of a coagulation-potentiating peptide LCTx-F2 on ASIC3 and ASIC1a channels. Through peptide mutation and electrophysiological recording, we identified the specific residues in LCTx-F2 that influence its binding affinity to these two channels. Moreover, molecular docking and dynamic simulation revealed that LCTx-F2 occupied the acidic pocket of its receptors, and the peptide interacted with the channels in distinct orientations. In addition, we examined the bonding relationship between the critical residues of LCTx-F2 and its receptors in this text. In summary, our findings demonstrate how the spider-derived peptide LCTx-F2 acts on ASICs and sheds light on the molecular mechanism underlying its inhibition of ASIC3 and ASIC1a channels. This provides a novel template for developing drugs that target these two channels.

## Experimental procedures

### Recombinant expression, purification, and identification of peptides

Peptide LCTx-F2 and its mutants were obtained by prokaryotic expression as we did before (38). Briefly, plasmids of LCTx-F2 or its mutants were transfected into *Escherichia coli* (DE3) cells, and protein expression was induced by IPTG in 28 °C for 12 h. After ultrasonication, the supernatant was purified by His tag and digested by tobacco etch virus protease. The peptides LCTx-F2 and its mutants were collected by reversed-phase HPLC (C4 column, 10 mm × 250 mm), and the molecular weight was identified by matrix-assisted laser desorption/ionization-time of flight mass spectrometry (Applied Biosystems).

### Electrophysiological recording

The plasmids containing the sequence of rASIC3 or ASIC1a channel were from Ye Yu lab in China Pharmaceutical University. The plasmid was transiently transfected into human embryonic kidney 293 cells following the protocol of Lipofectamine 2000 (CAS number: 12566014). After 24 h, whole-cell patch-clamp recordings were conducted using a MultiClamp 700B amplifier controlled by pCLAMP software (HEKA) in room temperature, as we did before (51). The extracellular solution contains (millimolar): 140 NaCl, 5 KCl, 2 MgCl_2_·6H_2_O, 10 d-glucose, 2 CaCl_2_·2H_2_O, 10 MES for pH 6.0, or 10 Hepes for pH 7.4, and pH was adjusted with NaOH. The intracellular solution contains (millimolar): 140 KCl, 5 NaCl, 2 MgCl_2_·6H_2_O, 10 d-glucose, 2 CaCl_2_·2H_2_O, 5 EGTA, 10 Hepes, and pH was adjusted with NaOH. All regents were purchased from Sigma–Aldrich company.

### Molecular docking and dynamic simulation

The molecular docking and dynamic simulation were performed as we did before (38, 51), using the predicted LCTx-F2 structure and the aligned rASIC3 channel structure model. The molecular docking was carried out using Discovery Studio 2019, with the parameters of angular step size 6^◦^, interface cutoff 9.0 Å, RMSD cutoff 6.0 Å, and maximum number of clusters 60 in all ZDOCK docking. Specially, the residues Arg7 and Asp10 in LCTx-F2 were set as the key sites for LCTx-F2 binding to rASIC3 channel structure model, and the residues Arg7 and Asp14 were for LCTx-F2 binding to rASIC1a channel. Virtual alanine scanning was performed in Discovery Studio 2019. The default cutoff value in the abovementioned protocol for categorizing mutations as neutral or destabilizing (in term of binding affinity to the ligand) is 0.5 kcal/mol as listed in BIOVIA Discovery Studio software. Three poses for LCTx-F2 binding to rASIC3 channel and one pose for LCTx-F2 binding to rASIC1a channel were chosen for dynamic simulation by GROMACS 2022. The complexes of LCTx-F2 binding to rASIC1a channel or rASIC3 channel were embedded in a water box containing 0.15 M NaCl, in which the protein charges were neutralized with Na^+^ or Cl^−^ ions. The GROMACS 2022 was used to the dynamic simulation with the CHARMN36m force field. After the equilibration, the simulation was carried out for 100 ns with a 2-fs time per step. The temperature at 303.15 K and the pressure at 1 bar were kept by the V-rescale and Parrinello–Rahman pressure control, representatively. The short-range electrostatic interactions were calculated by 12-Å cutoff, and the long-range electrostatic interactions were accounted based on the particle mesh Ewald summation method. Visual molecular dynamics was employed to analyze the dynamic simulation trajectories of all poses containing salt bridges, with the cutoff as 3.2 Å of oxygen–nitrogen distance, 3 Å of donor–acceptor distance, and 20 Å of angle.

## Data availability

The data that support the findings of this study are available on request from the corresponding author.

## Conflict of interest

The authors declare that they have no conflicts of interest with the contents of this article.

## References

[1] Deval E., Gasull X., Noël J., Salinas M., Baron A., Diochot S. (2010). Acid-sensing ion channels (ASICs): pharmacology and implication in pain. Pharmacol. Ther..

[2] Grunder S., Pusch M. (2015). Biophysical properties of acid-sensing ion channels (ASICs). Neuropharmacology.

[3] Huang Y., Jiang N., Li J., Ji Y.H., Xiong Z.G., Zha X.m. (2015). Two aspects of ASIC function: synaptic plasticity and neuronal injury. Neuropharmacology.

[4] Lin S.H., Sun W.H., Chen C.C. (2015). Genetic exploration of the role of acid-sensing ion channels. Neuropharmacology.

[5] Redd M.A., Scheuer S.E., Saez N.J., Yoshikawa Y., Chiu H.S., Gao L. (2021). Therapeutic inhibition of acid-sensing ion channel 1a recovers heart function after ischemia-reperfusion injury. Circulation.

[6] Kreple C.J., Lu Y., LaLumiere R.T., Wemmie J.A. (2014). Drug abuse and the simplest neurotransmitter. ACS Chem. Neurosci..

[7] Chen C.C., Wong C.W. (2013). Neurosensory mechanotransduction through acid-sensing ion channels. J. Cell Mol. Med..

[8] Harmata G.I.S., Chan A.C., Merfeld M.J., Taugher-Hebl R.J., Harijan A.K., Hardie J.B. (2023). Intoxicating effects of alcohol depend on acid-sensing ion channels. Neuropsychopharmacology.

[9] Kreple C.J., Lu Y., Taugher R.J., Schwager-Gutman A.L., Du J., Stump M. (2014). Acid-sensing ion channels contribute to synaptic transmission and inhibit cocaine-evoked plasticity. Nat. Neurosci..

[10] Zhu S., Zhou H.Y., Deng S.C., Deng S.J., He C., Li X. (2017). ASIC1 and ASIC3 contribute to acidity-induced EMT of pancreatic cancer through activating Ca(2+)/RhoA pathway. Cell Death Dis..

[11] Zhang Y., Liang J., Cao N., Gao J., Xie Y., Zhou S. (2022). ASIC1alpha up-regulates MMP-2/9 expression to enhance mobility and proliferation of liver cancer cells *via* the PI3K/AKT/mTOR pathway. BMC Cancer.

[12] Wemmie J.A., Taugher R.J., Kreple C.J. (2013). Acid-sensing ion channels in pain and disease. Nat. Rev. Neurosci..

[13] Sherwood T.W., Frey E.N., Askwith C.C. (2012). Structure and activity of the acid-sensing ion channels. Am. J. Physiol. Cell Physiol..

[14] Sun D., Yu Y., Xue X., Pan M., Wen M., Li S. (2018). Cryo-EM structure of the ASIC1a-mambalgin-1 complex reveals that the peptide toxin mambalgin-1 inhibits acid-sensing ion channels through an unusual allosteric effect. Cell Discov..

[15] Dawson R.J., Benz J., Stohler P., Tetaz T., Joseph C., Huber S. (2012). Structure of the acid-sensing ion channel 1 in complex with the gating modifier Psalmotoxin 1. Nat. Commun..

[16] Vullo S., Bonifacio G., Roy S., Johner N., Bernèche S., Kellenberger S. (2017). Conformational dynamics and role of the acidic pocket in ASIC pH-dependent gating. Proc. Natl. Acad. Sci. U. S. A..

[17] Yoder N., Yoshioka C., Gouaux E. (2018). Gating mechanisms of acid-sensing ion channels. Nature.

[18] Baconguis I., Bohlen C.J., Goehring A., Julius D., Gouaux E. (2014). X-ray structure of acid-sensing ion channel 1-snake toxin complex reveals open state of a Na(+)-selective channel. Cell.

[19] Baconguis I., Gouaux E. (2012). Structural plasticity and dynamic selectivity of acid-sensing ion channel-spider toxin complexes. Nature.

[20] Gonzales E.B., Kawate T., Gouaux E. (2009). Pore architecture and ion sites in acid-sensing ion channels and P2X receptors. Nature.

[21] Vullo S., Kellenberger S. (2020). A molecular view of the function and pharmacology of acid-sensing ion channels. Pharmacol. Res..

[22] Kellenberger S., Schild L. (2015). International Union of Basic and Clinical Pharmacology. XCI. structure, function, and pharmacology of acid-sensing ion channels and the epithelial Na+ channel. Pharmacol. Rev..

[23] Yu Y., Chen Z., Li W.G., Cao H., Feng E.G., Yu F. (2010). A nonproton ligand sensor in the acid-sensing ion channel. Neuron.

[24] Verkest C., Salinas M., Diochot S., Deval E., Lingueglia E., Baron A. (2022). Mechanisms of action of the peptide toxins targeting human and rodent acid-sensing ion channels and relevance to their *in vivo* analgesic effects. Toxins (Basel).

[25] Sun D., Liu S., Li S., Zhang M., Yang F., Wen M. (2020). Structural insights into human acid-sensing ion channel 1a inhibition by snake toxin mambalgin1. Elife.

[26] Diochot S., Baron A., Salinas M., Douguet D., Scarzello S., Dabert-Gay A.S. (2012). Black mamba venom peptides target acid-sensing ion channels to abolish pain. Nature.

[27] Bohlen C.J., Chesler A.T., Sharif-Naeini R., Medzihradszky K.F., Zhou S., King D. (2011). A heteromeric Texas coral snake toxin targets acid-sensing ion channels to produce pain. Nature.

[28] Pignataro G., Simon R.P., Xiong Z.G. (2007). Prolonged activation of ASIC1a and the time window for neuroprotection in cerebral ischaemia. Brain.

[29] Siemens J., Zhou S., Piskorowski R., Nikai T., Lumpkin E.A., Basbaum A.I. (2006). Spider toxins activate the capsaicin receptor to produce inflammatory pain. Nature.

[30] Osteen J.D., Herzig V., Gilchrist J., Emrick J.J., Zhang C., Wang X. (2016). Selective spider toxins reveal a role for the Nav1.1 channel in mechanical pain. Nature.

[31] Zhou X., Ma T., Yang L., Peng S., Li L., Wang Z. (2020). Spider venom-derived peptide induces hyperalgesia in Na(v)1.7 knockout mice by activating Na(v)1.9 channels. Nat. Commun..

[32] Grishin E.V., Savchenko G.A., Vassilevski A.A., Korolkova Y.V., Boychuk Y.A., Viatchenko-Karpinski V.Y. (2010). Novel peptide from spider venom inhibits P2X3 receptors and inflammatory pain. Ann. Neurol..

[33] Yuan F.C., Sun F.D., Zhang L., Huang B., An H.L., Rong M.Q. (2022). General mechanism of spider toxin family I acting on sodium channel Nav1.7. Zool Res..

[34] Zhang Y.X., Peng D.Z., Zhang Q.F., Huang B., Yang Q.C., Tang D.F. (2019). micro-TRTX-Ca1a: a novel neurotoxin from Cyriopagopus albostriatus with analgesic effects. Acta Pharmacol. Sin..

[35] McCarthy C.A., Rash L.D., Chassagnon I.R., King G.F., Widdop R.E. (2015). PcTx1 affords neuroprotection in a conscious model of stroke in hypertensive rats *via* selective inhibition of ASIC1a. Neuropharmacology.

[36] Chassagnon I.R., McCarthy C.A., Chin Y.K.Y., Pineda S.S., Keramidas A., Mobli M. (2017). Potent neuroprotection after stroke afforded by a double-knot spider-venom peptide that inhibits acid-sensing ion channel 1a. Proc. Natl. Acad. Sci. U. S. A..

[37] Li P., Zhang Z., Liao Q., Meng E., Mwangi J., Lai R. (2020). LCTX-F2, a novel potentiator of coagulation factors from the spider venom of Lycosa singoriensis. Front. Pharmacol..

[38] Yuan F., Li S., Huang B., Hu Y., Zeng X., Peng Y. (2023). Molecular mechanism by which spider-driving peptide potentiates coagulation factors. Biomed. Pharmacother..

[39] Stephan G., Huang L., Tang Y., Vilotti S., Fabbretti E., Yu Y. (2018). The ASIC3/P2X3 cognate receptor is a pain-relevant and ligand-gated cationic channel. Nat. Commun..

[40] Sun W.H., Chen C.C. (2016). Roles of proton-sensing receptors in the transition from acute to chronic pain. J. Dent Res..

[41] Chen C.C., Zimmer A., Sun W.H., Hall J., Brownstein M.J., Zimmer A. (2002). A role for ASIC3 in the modulation of high-intensity pain stimuli. Proc. Natl. Acad. Sci. U. S. A..

[42] Dulai J.S., Smith E.S.J., Rahman T. (2021). Acid-sensing ion channel 3: an analgesic target. Channels (Austin).

[43] Osmakov D.I., Kozlov S.A., Andreev Y.A., Koshelev S.G., Sanamyan N.P., Sanamyan K.E. (2013). Sea anemone peptide with uncommon beta-hairpin structure inhibits acid-sensing ion channel 3 (ASIC3) and reveals analgesic activity. J. Biol. Chem..

[44] Lee J.Y.P., Saez N.J., Cristofori-Armstrong B., Anangi R., King G.F., Smith M.T. (2018). Inhibition of acid-sensing ion channels by diminazene and APETx2 evoke partial and highly variable antihyperalgesia in a rat model of inflammatory pain. Br. J. Pharmacol..

[45] Kuhn-Nentwig L., Fedorova I.M., Lüscher B.P., Kopp L.S., Trachsel C., Schaller J. (2012). A venom-derived neurotoxin, CsTx-1, from the spider Cupiennius salei exhibits cytolytic activities. J. Biol. Chem..

[46] Oparin P.B., Nadezhdin K.D., Berkut A.A., Arseniev A.S., Grishin E.V., Vassilevski A.A. (2016). Structure of purotoxin-2 from wolf spider: modular design and membrane-assisted mode of action in arachnid toxins. Biochem. J..

[47] Heusser S.A., Borg C.B., Colding J.M., Pless S.A. (2022). Conformational decoupling in acid-sensing ion channels uncovers mechanism and stoichiometry of PcTx1-mediated inhibition. Elife.

[48] Luo L., Li B., Wang S., Wu F., Wang X., Liang P. (2018). Centipedes subdue giant prey by blocking KCNQ channels. Proc. Natl. Acad. Sci. U. S. A..

[49] Du C., Li J., Shao Z., Mwangi J., Xu R., Tian H. (2019). Centipede KCNQ inhibitor SsTx also targets KV1.3. Toxins (Basel).

[50] Garcia-Fernandez R., Peigneur S., Pons T., Alvarez C., González L., Chávez M.A. (2016). The Kunitz-type protein ShPI-1 inhibits serine proteases and voltage-gated potassium channels. Toxins (Basel).

[51] Du C., Guan X., Yan J. (2022). Two-pore channel blockade by phosphoinositide kinase inhibitors YM201636 and PI-103 determined by a histidine residue near pore-entrance. Commun. Biol..

